# Secretome Survey of Human Plexiform Neurofibroma Derived Schwann Cells Reveals a Secreted form of the RARRES1 Protein

**DOI:** 10.3390/ijms13079380

**Published:** 2012-07-24

**Authors:** Hui-Ling Chen, Haeri Seol, Kristy Jean Brown, Heather Gordish-Dressman, Ashley Hill, Vittorio Gallo, Roger Packer, Yetrib Hathout

**Affiliations:** Children’s National Medical Center, 111 Michigan Avenue NW, Washington, DC 20010, USA; E-Mails: hchen@cnmcresearch.org (H.-L.C.); HSeol@cnmcresearch.org (H.S.); KBrown@cnmcresearch.org (K.J.B.); HGordish@cnmcresearch.org (H.G.-D.); dashill@cnmc.org (A.H.); VGallo@cnmcresearch.org (V.G.); RPACKER@cnmc.org (R.P.)

**Keywords:** plexiform neurofibroma, Schwann cells, secretome, retinoic acid receptor responder protein1

## Abstract

To bring insights into neurofibroma biochemistry, a comprehensive secretome analysis was performed on cultured human primary Schwann cells isolated from surgically resected plexiform neurofibroma and from normal nerve tissue. Using a combination of SDS-PAGE and high precision LC-MS/MS, 907 proteins were confidently identified in the conditioned media of Schwann cell cultures combined. Label free proteome profiling revealed consistent release of high levels of 22 proteins by the four biological replicates of NF1 Schwann cell cultures relative to the two normal Schwann cell cultures. Inversely, 9 proteins displayed decreased levels in the conditioned media of NF1 relative to normal Schwann cells. The proteins with increased levels included proteins involved in cell growth, angiogenesis and complement pathway while proteins with decreased levels included those involved in cell adhesion, plasminogen pathway and extracellular matrix remodeling. Retinoic acid receptor responder protein-1 (RARRES1), previously described as an integral membrane tumor suppressor, was found exclusively secreted by NF1 Schwann cells but not by normal Schwann cells. All-trans retinoic acid modulated secretion of RARRES1 in a dose dependent manner. This study shows altered secretion of key proteins in NF1 derived Schwann cells. The potential implication of these proteins in neurofibroma biology is discussed.

## 1. Introduction

Plexiform neurofibromas are benign peripheral nerve sheath tumors affecting 26 to 30% of patients diagnosed with neurofibromatosis type I (NF1) [1]. They are often present as large peripheral nerve neoplasms affecting the head, neck and/or lower back [2]. They are ultimately composed of uncharacterized cell type of the Schwann cell lineage origin intertwined with fibroblasts, mast cells and endothelial cells [3]. Though benign, these peripheral nerve tumors can be clinically debilitating depending on their size, location and infiltrative nature [4]. Furthermore, individuals with plexiform neurofibroma are at risk of developing malignant peripheral nerve sheath tumors (MPNSTs) which are more challenging to treat. The only effective treatment available today for plexiform neurofibroma is surgical resection [4,5]. Unfortunately, depending on the location and the infiltrative nature of the tumor masses, complete surgical resection remains a challenge often leading to recurrence of even larger tumor masses causing pain, disfigurement and physical impairment [5,6].

It is suggested that plexiform neurofibroma arise from Schwann cells that undergo loss of heterozygosity at the *NF1* locus [7]. Schwann cells are of neural crest origin and play an important role in the development and maintenance of the peripheral nervous system [8]. They are involved in the myelination and insulation of neuronal axons as well as in the regeneration and trophic support for neurons. During the course of NF1 pathogenesis Schwann cells undergo biallelic inactivation at the *NF1* locus leading to somatic inactivation of NF1 gene that encode for neurofibromine-1 protein, a physiological inhibitor of the Ras pathway [1,9]. This results in hyper-activation of the Ras pathway and uncontrolled proliferation of Schwann cells.

Agents targeting the Ras pathway and kinase pathways showed promise in inhibiting neurofibroma progression *in vitro* and in mouse models [10]. Unfortunately, these same agents had little to no impact on patients with progressive plexiform neurofibroma indicating that Ras pathway alone does not account for the plexiform neurofibroma progression [11].

Recent studies suggested involvement of the cell microenvironment and cell-cell interaction in neurofibroma progression [12,13]. Proteins secreted by both Schwann cells and mast cells might contribute to the uncontrolled growth of neurofibroma. Thus, a comprehensive study of secreted proteins or “secretome” of the implicated cells might be an attractive approach for identifying components that might bring insight into the molecular pathogenesis of NF1.

In this study, we first sought to define the secretome of primary Schwann cell cultures derived from surgically resected plexiform neurofibroma. We used a label free proteome profiling strategy to systematically compare secretomes of four plexiform neurofibroma Schwann cell cultures to the secretome of normal Schwann cells derived from non-neoplastic peripheral nerve. This survey identified aberrant release of several key proteins by the plexiform neurofibroma Schwann cells relative to the normal Schwann cells. Retinoic acid receptor responder protein 1 (RARRES1) also known as Tazarotene-induced gene (TIG1) was exclusively released by the plexiform neurofibroma Schwann cells and not by normal Schwann cells derived from non-noeplastic peripheral nerve.

Identifying altered protein secretion by neurofiboma Schwann cells might shed light into the mechanism of neurofibroma progression and eventually define novel therapeutic targets for NF1 patients suffering from recurrent plexiform neurofibroma.

## 2. Results and Discussion

### 2.1. Primary Schwann Cell Isolation and Culture

**Figure 1:**
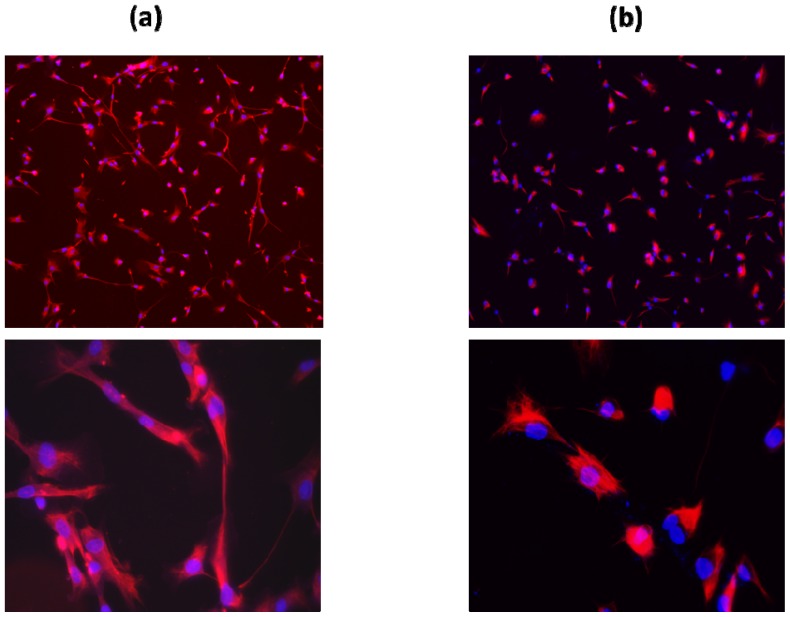
Human primary Schwann cell cultures maintained *in vitro* for 2 weeks in basal Schwann cell media. (**a**) Normal Schwann cells; (**b**) Schwann cells isolated from a plexiform neurofibroma of a 20 year old female donor (NFSC 141). Both cells were immuno-stained against the Schwann cell marker S100β protein shown in red and the nuclei with DAPI shown in blue.

NFSC141, NFSC142, NFSC143R and NFSC143L primary Schwann cells cultures were established from plexiform neurofibroma surgically resected from three different patients admitted at CNMC. NFSC143R and NFSC143L were established from tumors removed from the right and left lumbar of the same patient. Table 1 summarizes the age, gender and tumor location of the participating NF1 patients. All three patients were diagnosed with a plexiform type of neurofibroma according to the pathology results. Established Schwann cell cultures from these specimens showed highly homogenous cell population. All NF1 Schwann cultures resembled the normal Schwann cell culture and expressed the characteristic Schwann cell marker S100β (Figure 1). The isolated cells were further subcultured to passage 2 and processed for secretome profiling and Western blot analysis and the results are described below.

### 2.2. Secretome Profiling

Secretome profiles of the four plexiform neurofibroma Schwann cell cultures NFSC141, NFSC142, NFSC143R and NFSC143L and the two normal primary human Schwann cell cultures NSC1 and NSC2 were obtained as described in the method using a combination of SDS-PAGE, in-gel digestion and high precision LC-MS/MS. We confidently identified and quantified 907 proteins in the CM of the four NF1 Schwann cell cultures and the two normal Schwann cell cultures combined (see Supplemental Table 1). Only proteins that were identified by at least two unique peptides and probability based score p value < 0.001 were retained for further analysis. In general Schwann cells were found to secrete a variety of extracellular matrix remodeling proteins, complement components, cell growth regulating proteins, cell adhesion and cell migration promoting proteins.

For quantitative comparisons we used ProteoIQ software to compare spectral counts and signal intensities generated from peptides belonging to each protein across all the samples analyzed. Several proteins were found uniquely secreted by NF1 Schwann cells and not by the normal Schwann cells and vice versa. Among these differentially secreted proteins, 12 were exclusively secreted by the four NF1 Schwann cell cultures and not by the normal Schwann cells, while 4 were exclusively secreted by the normal Schwann cells but not by any of the NF1 Schwann cell cultures. These proteins are reported in Table 2 with their spectral count, subcellular localization and potential function. NF1 Schwann cells secreted significant amounts of complement components C3 and C4-A, retinoic acid receptor responder protein1 (RARRES1), apolipoproteins D and E, Chitinase-3 like protein and glutathione peroxidase 3. The RARRES1 protein was among the most abundant proteins in this category with average spectral counts of 45 versus 0 in the CM of NF1 Schwann cells relative to normal Schwann cells.

Other proteins were secreted by both NF1 and normal Schwann cells but in different amounts. Secretion of 7 proteins were found to be increased by at least 3 fold with *p* value ≤ 0.05 while 6 were found to be decreased by at least the same factor and p value cut off in CM of NF1 Schwann cells relative to normal Schwann cells. These proteins are reported in Table 3 with their spectral counts, subcellular localization and potential function.

Overall, plexiform neurofibroma Schwann cells released greater amounts of cell adhesion proteins such as laminin alpha 4, nidogen-2, cell growth regulating factors such as target of Nesh-SH3, EGF-containing fibulin-like extracellular matrix protein 1 and insulin-like growth factor binding proteins while dramatically decreased the release of receptor type tyrosine protein phosphatase kappa, a negative regulator of EGFR signaling as well as, plasminogen activator inhibitor 1 (PAI-1), chondroitin sulfate proteoglycan 4 (CSPG4), and integrin beta-1.

The altered secretome signature in NF1 Schwann cells relative to normal Schwann cells was maintained in the commercially available sNF96.2 cell line established from a malignant peripheral nerve sheath tumor that was surgically resected from a 27 year old donor five years ago [14] (see supplemental Table). Several proteins that were elevated or decreased in the CM of NF1 Schwann cells relative to normal Schwann cells maintained the same expression direction in the CM of sNF96.2 cells relative to normal Schwann cells. These include the RARRES1 protein, Complements, chitinase 3-like protein, alpha fetoprotein, cadherin-6 *etc*. (see supplemental Table)

### 2.3. Characterization of Schwann Cell Secreted RARRES1 Protein

**Figure 2:**
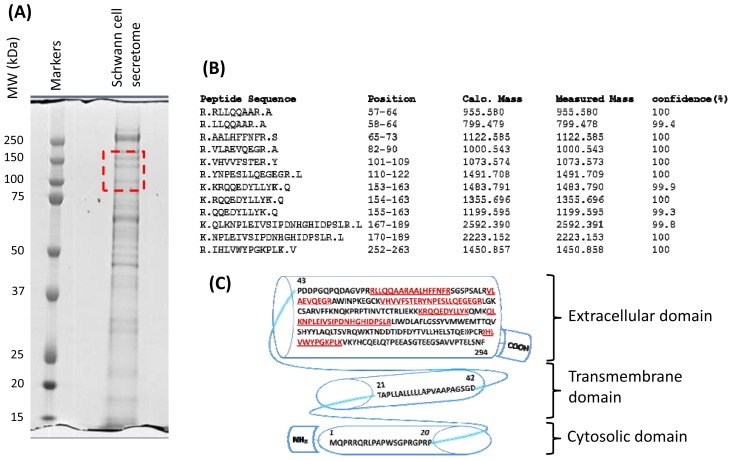
Peptide mapping for the Schwann cell secreted RARRES1. (**A**) SDS-PAGE of total proteins collected in the conditioned medium of a primary NF-1 Schwann cell culture. The boxed area in the gel indicates where RARRES1 protein was detected by mass spectrometry analysis; (**B**) List of detected and identified tryptic peptides for the secreted RARRES1 protein with their expected and measured molecular masses and sequencing confidence; (**C**) Primary sequence of the RARRES1 protein with N-terminal cytosolic domain, integral membrane domain and the C-terminal extracellular domain. Detected and identified tryptic peptides for the secreted RARRES1 are highlighted in red and underlined. No tryptic peptide was detected for the integral membrane and the cytosolic domain.

An earlier study described RARRES1 protein as a 33 kDa single pass trans-membrane protein with a short N-terminal cytosolic domain, a transmembrane domain and a large C-terminal [15]. The secreted RARRES1 was identified by 12 unique tryptic peptides in SDS-PAGE bands ranging from 80 to 160 kDa with higher concentration around 110 kDa. This apparent mass is much higher than the expected 38–40 kDa molecular mass reported for the cell attached RARRES1 protein [15,16]. The commercially available sNF96.2 Schwann cell line established from MPNST also secreted similar high molecular mass RARRES1 protein. The sequences of the detected peptides for the extracellular RARRES1 and their position in the protein are shown in Figure 2. The observed masses for these peptides were within 10 ppm accuracy with confidence identification better than 99%. All 12 peptides belonged to the C-terminal extracellular domain of the protein suggesting possible shedding of RARRES1 from NF1 Schwann cells or secretion of a novel splice isoform. Interestingly, the commercially available rabbit polyclonal antibodies (from two different sources and with different target epitope) raised against cell attached RARRES1 failed to detect the secreted form of RARRES1 (data not shown). This result suggests a potential posttranslational modification of the secreted RARRES1.

### 2.4. mRNA RARRES Expression Levels in NF1 *Versus* Normal Schwann Cells

#### 2.4.1. Quantitative RTPCR

**Figure 3:**
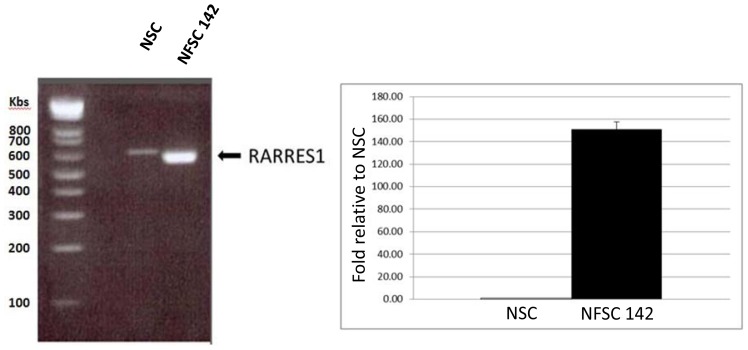
RTPCR analysis of the RARRES1 mRNA expression in NF1 and normal Schwann cell pellets. Left panel shows the RTPCR product obtained on mRNA prepared from normal Schwann cell culture (NSC) and plexiform neurofibroma Schwann cell culture (NFSC 142). Right panel shows the relative quantities of the RARRES1 mRNA levels in normal Schwann cells (NSC) and Schwann cells isolated from a plexiform neurofibroma of a 19 year old male (NFSC 142). Values are normalized to internal control GAPDH mRNA levels.

Quantitative RTPCR targeting RARRES1 mRNA in total cell lysates showed a 150 fold higher level of RARRES1 expression in NF1 Schwann cells relative to normal Schwann cells (Figure 3). This data shows that the RARRES1 gene was actively expressed in plexiform neurofibroma Schwann cells compared to normal Schwann cells and correlates with the high levels of secreted RARRES1 protein seen in NF1 Schwann cells relative to normal Schwann cells.

#### 2.4.2. Western Blot Analysis

**Figure 4:**
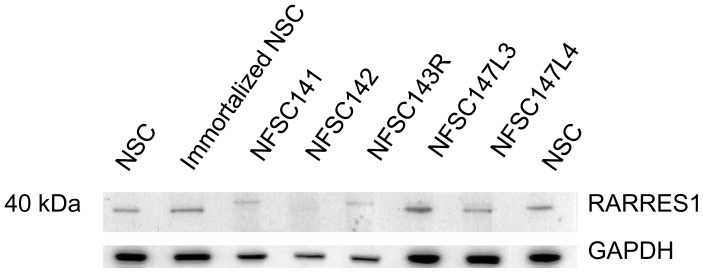
Expression levels of cell attached RARRES1 in normal versus NF1 Schwann cells. Cell pellets were prepared from two normal Schwann cell culture (NSC and immortalized NSC) and five plexiform neurofibroma Schwann cell cultures (NFSCs). Expression levels of cell attached RARRES1 was examined in cell pellets using commercially available antibody. Lower panel show Western blot of a loading control GAPDH protein.

To verify if this high expression of RARRES1 mRNA in NF1 Schwann cells relative to normal Schwann cells translates into increased expression of the RARRES1 protein in the cell pellets we performed Western blot analysis on the total protein extracts from NF1 and normal Schwann cells using two different rabbit polyclonal antibodies against human RARRES1 protein. Unlike the secreted RARRES1, the cell attached form of the RARRES1 was detected as a single band around 40 kDa in agreement with the antibody manufacturer datasheet and with earlier studies [16]. However, there was no significant difference in the expression levels of the 40 kDa RARRES1 between the normal and NF1 Schwann cells (Figure 4).

### 2.5. All-Trans Retinoic Acid Induces Expression of Secreted RARRES1

**Figure 5:**
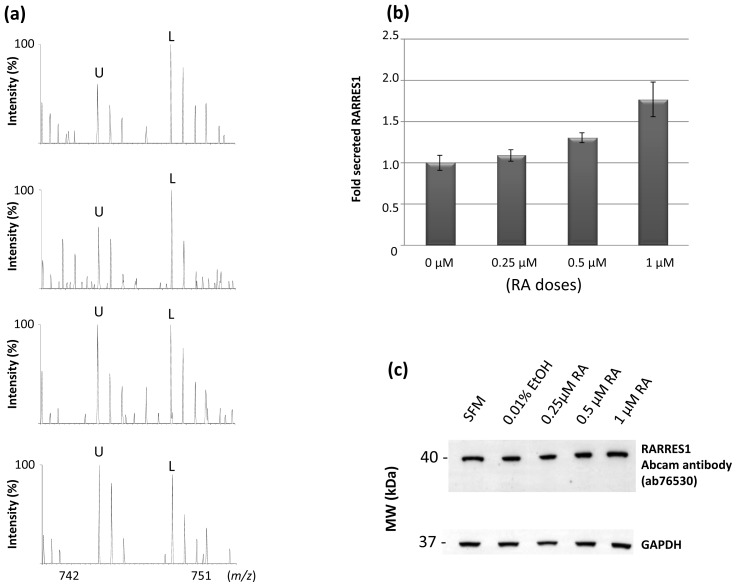
Targeted mass spectrometry quantification of the secreted RARRES1 by sNF96.2 cells treated with different doses of all-trans retinoic acid. (**a**) MS spectra of doubly charged ions at *m/z* 758.92 and 761.92 for the unlabeled (U) and labeled (L) peptide (YNPESLLQEGEGR) generated from tryptic digest of the *de novo* synthesized RARRES1 and the spiked-in labeled standard RARRES1 respectively. Spectra from the top to bottom are recorded for the same peptide pairs in function of different RA doses (0, 0.25, 0.5 and 1 μM). The intensity of the unlabeled peptide increases with increased doses of RA; (**b**) Bar graphs of the relative expression of *de novo* secreted RARRES1 in function of different RA concentrations. Standard deviations were obtained from measuring 4 unique peptide sequences of the RARRES1 protein (*p*-values < 0.05); (**c**) Western blot for the cell attached RARRES1 protein in function of RA treatment using a rabbit polyclonal antibody against human RARRES1 protein detecting a band around 40 kDa. Note that while the extracellular RARRES1 increased by almost a factor of 2 following treatment with 1 μM of RA for 24 hours the cellular attached RARRES1 remained unchanged.

Since the commercially available RARRES1 antibodies failed to detect extracellular RARRES1 we synthesized a stable isotope labeled RARRES1 standard by culturing sNF96.2 cell line in medium where Arg and Lys were replaced by stable isotope labeled ^13^C_6_-Arg and ^13^C_6_, ^15^N_2_-Lys. All secreted proteins including RARRES1 protein were uniformly and fully labeled with ^13^C_6_-Arg and ^13^C_6_, ^15^N_2_-Lys after 7 cell doublings. A fixed amount of the stable isotope labeled RARRES1 was then spiked into equal aliquots of conditioned media collected from unlabeled sNF96.2 cells treated for 24 hours with 0, 0.25, 0.5 or 1 μM RA of all-trans retinoic acid (RA). Spiked samples were processed for mass spectrometry analysis as described in the method. Peptides generated from *de novo* synthesized RARRES1 protein and the labeled spiked-in RARRES1 proteins are detected as pairs of light and heavy ions. Figure 5a shows example spectra of heavy and light ion pairs corresponding to labeled and unlabeled RARRES1 tryptic peptide (YNPESLLQEGEGR) generated from labeled standard RARRES1 and *de novo* synthesized RARRES1. Figure 5b shows the overall expression levels of the cellular secreted RARRES1 by sNF96.2 treated with different doses of RA (the levels are relative to the spiked-in SILAC labeled standard RARRES1). While the expression levels of secreted RARRES1 increased in a dose dependent manner in response to RA treatment with almost a 2-fold increase after 24 hours incubation with 1 μM RA dose, the band detected by Western blot analysis for the cell attached RARRES1 remained unchanged following treatment of sNF96.2 cells with RA even at a higher dose of 1 μM of RA (Figure 5c).

### 2.6. Discussion

Secretome profiling of human primary Schwann cell cultures revealed several key proteins that were differentially secreted between the plexiform neurofibroma derived Schwann cells and the non-neoplastic peripheral nerve derived Schwann cells. Most of these proteins are known to play a direct role in cell adhesion, cell proliferation and tumorigenesis. These include the target of Nesh-SH3 protein also known as TARSH. This protein was approximately 19-fold higher in the CM of NF1 Schwann cells relative to normal Schwann cells. TARSH was recently described to play a role in cell senescence via the p53 pathway [17] but also play a major role in reducing cell dendritic complexity of neural cells [18]. This later function might be relevant to the differentiation of neural and Schwann cells in the context of neurofibroma progression. Further studies to define the role of TARSH in growth and differentiation of Schwann cells are needed.

APOD was exclusively secreted by all four NF1 Schwann cells cultures and not by normal Schwann cells. This is in agreement with earlier studies showing that APOD mRNA levels were increased in neurofibroma compared to non-neoplastic peripheral nerve [19]. Interestingly, the same group showed that APOD mRNA levels decreases during transformation of benign neurofibroma to MPNSTs. The exact role of APOD in neurofibroma progression is however not well understood at present. Nevertheless expression of APOD was often found to be increased in soft tissue tumors and could as well have a direct role in growth of benign tumors [20].

Alpha-fetoprotein (AFP) is another interesting protein that was significantly elevated in the conditioned media of the four NF1 Schwann cell cultures compared to the normal Schwann cell cultures. AFP is a 70 kDa glycoprotein that is normally expressed during fetal development then repressed after birth [21]. Its reappearance often indicates the presence of a tumor especially hepatocellular carcinoma [22] but also other tumors of germ cell origin and gastrointestinal tract [23,24]. Furthermore, AFP was reported to promote proliferation of NIH 3T3 cells via activation of the Ras pathway [25]. These data suggests that AFP might be involved in the growth and proliferation of NF1 Schwann cells by acting in synergy with the inactive neurofibormin-1 thus resulting in hyperactivation of the Ras pathway. However, this hypothesis need to be further verified using mouse knockdown assays.

The dramatic decrease of PAI-1 in the extracellular milieu of NF1 Schwann cells relative to normal Schwann cells is also highly significant in the context of neurofibroma growth and progression. Indeed, PAI-1 acts as a physiological inhibitor of urokinase-type plasminogen activator (uPA), a serine protease that promotes tumor progression and invasiveness [26]. An earlier study targeting the plasminogen activation pathway in cutaneous neurofibroma demonstrated that Schwann cells, and not other cells, in the neurofibroma tissue are responsible for the release of uPA and its regulator PAI-1 [27]. In this study we were not able to detect uPA in the secretome profiles of normal and NF1 Schwann cells but we detected a significant amount of uPA (7 spectral counts) in the conditioned medium of the malignant sNF96.2 Schwann cell line (see supplemental Table). This is in agreement with the malignancy of the sNF96.2 relative to the NF1 and normal Schwann cells studied herein.

Other interesting proteins that were abundant in the extracellular milieu of NF1 Schwann cells relative to normal Schwann cells include the perlecan also known as basement membrane-specific heparan sulfate proteoglycan core protein, a secreted heparan sulfate glycoprotein involved in endothelial cell proliferation, mitogenesis and angiogenesis [28]. The high secretion of this protein by NF1 Schwann cell might contribute the complex growth and vasculature often seen in plexiform neurfibroma tissue. Furthermore NF1 Schwann cells secreted higher amounts of EFEMP1, an activator of the EGFR signaling [29] while decreasing the levels of the receptor-type tyrosine-protein phosphatase kappa, a negative regulator of EGFR signaling [30,31]. This suggests that EFGR signaling might be hyperactivated in NF1 Schwann cells relative to normal Schwann cells. However, this hypothesis needs to be verified in future studies.

Perhaps one of the most interesting findings in the secretome survey of Schwann cells is the exclusive secretion of a high molecular mass RARRES1 protein (~110 kDa) by the four NF1 Schwann cultures including the sNF96.2 cell line and not by the normal Schwann cell cultures. Secretion of RARRES1 protein is novel and to the best of our knowledge never been reported or discussed before. Whether the protein was proteolytically cleaved from the cell surface or was secreted as a splice isoform remains to be carefully examined. Nevertheless, all the RARRES1 tryptic peptides identified by mass spectrometry in the CM of NF1 Schwann cells belonged to the extracellular domain of the protein. No peptides of the N-terminal domain or the transmembrane domain were detected suggesting potential secretion or shedding of the RARRES1 protein.

The RARRES1 gene was first characterized by Nagpal *et al*. [15] as a target gene that was induced in human cultured skin tissue by a synthetic retinoid Tazarotene, hence the name Tazarotene-induced gene 1 (TIG-1). The gene was suggested to express a 294 amino acid transmembrane protein with molecular mass of 33,285 Da. From the sequence it was predicted that RARRES1 is a single pass type II membrane protein with a short cytosolic N-terminal domain, a transmembrane domain and large extracellular C-terminal domain. In this study we found RARRES1 protein to be actually secreted or released in the extracellular milieu by NF1 Schwann cells. Interestingly, while RARRES1 protein extracted from the cell pellet was detected as a single band around 40 kDa by Western blot, the RARRES1 protein released into the CM by NF1 Schwann cells was detected in multiple bands ranging from 80 to 160 kDa with a highest concentration around 110 kDa. The antibody used to detect cell attached RARRES1 protein failed to detect the secreted RARRES1 even though several peptides were detected by mass spectrometry for this protein in the CM of cultured NF1 Schwan cells. This suggests that the released RARRES1 protein might be post-translationally modified hindering its detection by the antibody that detects the 40 kDa cell attached RARRES1. Based on the Uniprot knowledge database, human RARRES1 protein has only one potential N-glycosylation site which is not enough to explain or support the high increase in the molecular mass (up to 160 kDa) seen for extracellular RARRES1. Even though RARRES1 protein was previously detected in the conditioned media of some cancer cell lines [32,33] no details were provided regarding its molecular mass and its occurrence in the extracellular milieu. In this study we consistently detect secreted RARRES1 in bands ranging from 80 to 160 kDa with the highest abundance around 110 kDa. All SDS-PAGE separations were performed under denaturing conditions thus ruling out the possibility of oligomerization. This significant increase in the molecular mass is typical for heparan sulfate modifications. However, future structural studies are needed to verify this hypothesis.

Secretion of the high molecular mass RARRES1 protein was maintained in the sNF96.2 Schwann cell line previously established from an MPNST neurofibroma that was surgically resected from a 27 year old donor five years ago [14]. We and others have examined secretomes of several human primary and tumor cell lines including retinal pigment epithelial cells [34,35], primary astrocytes, glioblastoma cell lines [36], twelve different types of human tumors cell lines [32], twenty three human carcinoma cells lines [33] and primary myotubes [37]. Of these cells only the Hela cell line, the colon cancer cell lines SW620 and Colo20 [33] and the lung carcinoma cell line A549 [32] secreted RARRES1 protein in their conditioned media. These data suggest that secretion of RARRES1 does occur and might be specific to certain cancer cell lines including our NF1 derived Schwann cells. Furthermore, close examination of the protein list identified in a secretome study of the omental adipose tissue explant, also revealed the presence of RARRES1 protein in the conditioned medium [38]. In another independent study high expression levels of RARRES1 gene, up to 59-fold was observed in slowly proliferating mesenchymal stem cells derived from visceral adipose tissue versus highly proliferative mesenchymal stem cells derived from subcutaneous adipose [39]. It was suggested that RARRES1 might be involved in regulating proliferation as well as differentiation of adipose tissue derived mesenchymal stem cells. However, the authors did not distinguish between secreted RARRES1 or cell attached RARRES1 since measurements were performed at the mRNA levels. In summary, these data on the secretome studies of cancer cell lines, the adipose tissue explant and our current Schwann cell secretome strongly support the existence of a secreted form of the RARRES1 protein. This secreted RARRES1 might play role in cell differentiation and growth.

While this secreted RARRES1 protein seems to be increased in certain cancer cell lines, the attached RARRES1 protein was often reported to be less expressed, at least at the mRNA level, in prostate carcinoma, nasopharyngeal carcinoma and a leukemia cell line [40–42]. Others have shown that RARRES1 protein expression changes in function of cancer stage, with high expression at early stage then down-regulated at advanced stage [43]. The down-expression was often attributed to hypermethylation of the promoter region of the *RARRES1* gene. In our study, even though the secreted RARRES1 was highly expressed in the NF1 Schwann cells relative to normal Schwann cells there was no significant difference in the expression levels of the 40 kDa cell attached RARRES1 between these cells. Additionally, treatment of sNF96.2 cells with increasing doses of all-trans retinoic resulted in increased secretion of the RARRES1 protein but not the 40 kDa cell attached RARRES1 protein. This suggests that these two forms of the RARRES1 protein might be posttranslationally regulated or originates from mRNA splicing that is tightly controlled in these Schwann cells. However this hypothesis remains to be carefully examined.

The lower expression of cell attached RARRES1 in prostate and nasopharyngeal carcinoma has suggested that RARRES1 protein might act as a tumor suppressor. However, mechanisms by which RARRES1 inhibits cell proliferation in cancer are largely unknown. Recent studies have shown that RARRES1 is a potential inhibitor of the cytoplasmic carboxypeptidase AGBL2, a protein that is involved in the regulation of tubulin tyrosination and tumorigenesis [44]. Furthermore the same group showed that knockdown of RARRES1 in human prostatic epithelial cell line resulted in down-regulation of an enzyme involved in negative regulation of the growth hormone-stimulated signal transduction pathways with concomitant up-regulation of a tumor suppressor disks large 2 [16]. While this might be true for the cell attached RARRES1 the over expression of secreted RARRES1 in NF1 Schwann cells and other cancer cell lines remains intriguing and requires further investigation to define the role of secreted RARRES1 protein in the context of tumor cells.

## 3. Experimental Section

### 3.1. Specimen Collection

Normal human primary Schwann cells were purchased from ScienCell Research Laboratories (Carlsbad, CA). The normal Schwann cells were isolated from spinal nerve of healthy donor according to the manufacturer and were cultured into two separate batches named as NSC1 and NSC2 herein. The malignant sNF96.2 Schwann cell line was purchased from the American Type Culture Collection (ATCC; Manassas, VA, USA). Immortalized normal human Schwann cells were a gift from Vedant Arun and Dr. Guha of the University of Toronto Canada. All neurofibroma specimens and nerve tissue specimens were collected in house in accordance with an Institutional Review Board approved protocol at Children’s National Medical Center (CNMC) and after obtaining patients’ written informed consent. Only excess specimens after diagnosis were used in this study. Diagnosis was performed at the pathology clinic at CNMC by Dr. Ashley Hill and reported to us as de-identified information where only age, gender, diagnosis and tumor location were included (Table 1). Normal sural nerve tissue was gift from Dr. Ashley Hill and was collected postmortem from a 9 week old female donor. Normal sciatic nerve was obtained from the NICHD Brain and Tissue Bank of the University of Maryland Medical School. Aliquots from freshly resected tumor specimens were immediately placed in a sterile solution of serum free DMEM medium for subsequent Schwann cells isolation and cultivation as described below.

### 3.2. Isolation and Culture of Human Primary Schwann Cells

Collected fresh tissue from each patient was dissected into 2 × 2 mm^2^ pieces and placed in a Petri Dish containing 10 mL of DMEM media supplemented with 0.8 U/mL of dispase (Sigma-Aldrich, St. Louis, MO, USA) and 160 U/mL of collagenase-1 (Sigma-Aldrich, St. Louis, MO, USA) followed by incubation at 37 °C for 2–4 h. Media was collected and dissociated cells were pelleted by gentle centrifugation at 300× g for 10 min. Pelleted cells were re-suspended and cultured in basal Schwann Cell Medium (SCM) from ScienCell Research Laboratories (Carlsbad, CA, USA) supplemented with 10% FBS, 100 U/mL penicillin, 100 μg/mL streptomycin and 1% Schwann cell growth supplement (SCGS) containing BSA 10 μg, insulin 5 μg, PDGF 5 ng, FGF-2 5 ng, progesterone 20 nM, forskolin 200 nM, T3 60 nM and PMA 20 nM. Growing cells were maintained and passaged using the same proprietary media. In parallel, primary human Schwann cells obtained from ScienCell Research Laboratories (Carlsbad, CA, USA) as well as sNF96.2 cell line were cultured using the same media and conditions. Growing Schwann cells were characterized by immunofluorescent staining using rabbit antibodies against human S-100β and p75 proteins.

### 3.3. Secretome Profiling

Both normal and plexiform neurofibroma derived Schwann cells as well as sNF96.2 cell line were grown to 80% confluences in the SCM media above then washed 6 times with sterile PBS (Invitrogen, Carlsbad, CA, USA) before incubation in serum free media for an additional 24 hours. Conditioned media (CM) were then collected, centrifuged at 300× g and the supernatant were further filtered through a 0.22 μm nylon filter (Millipore, Bedford, MA, USA) to remove any cell debris or floating cells. Aliquots (2 mL) from each collected CM were further concentrated by using a 3 kDa cutoff filter (Millipore Corp., Billerica, MA, USA) and processed for SDS-PAGE and mass spectrometry analysis. After the gel was stained (Bio-Safe Coomassie; Bio-Rad), each lane was sliced into 30 bands, and each band was in-gel digested with trypsin (Promega, Madison, WI, USA) and the resulting peptides analyzed by LC-MS/MS as described below.

### 3.4. Liquid Chromatography-Tandem Mass Spectrometry

Concentrated peptides from each band were injected via an autosampler (6 μL) and loaded onto a Symmetry C18 trap column (5 μm, 300 μm i.d. × 23 mm, Waters, Milford, MA, USA) for 10 min at a flow rate of 10 μL/min, 100% A. The sample was subsequently separated by a C18 reverse-phase column (3.5 μm, 75 μm × 15 cm, LC Packings, Sunnyvale, CA, USA) at a flow rate of 250 nL/min using an Eksigent nano-HPLC system (Dublin, CA, USA). The mobile phases consisted of water with 0.1% formic acid (A) and 90% acetonitrile (B). A 65 minute linear gradient from 5 to 60% B was employed. Eluted peptides were introduced into the mass spectrometer via a 10 μm silica tip (New Objective Inc., Ringoes, NJ, USA) adapted to a nano-electrospray source (Thermo Fisher Scientific). The spray voltage was set at 1.2 kV and the heated capillary at 200 °C. The LTQ-Orbitrap-XL (ThermoFisher Scientific) was operated in data-dependent mode with dynamic exclusion in which one cycle of experiments consisted of a full-MS in the Orbitrap (300–2000 m/z, resolution 30,000) survey scan and five subsequent MS/MS scans in the LTQ of the most intense peaks using collision-induced dissociation with the collision gas (helium) and normalized collision energy value set at 35%.

### 3.5. Database Search and Quantification

For protein identification we used the SEQUEST search engine [45] and for label free proteome profiling we used ProteoIQ software (Nusep, Bogart, GA, USA). Database search was performed against human SwissProt database (Version 198; EuropeanBioinformatics Institute: Cambridge, UK, 2012) indexed with assumptions for fully enzymatic tryptic cleavage with two missed cleavages and possible 16 Da shift for oxidized Met. The search result was filtered using the following acceptance criteria: DeltaCn (ΔCn) > 0.08, a variable threshold of Xcorr vs charge state (Xcorr ≥ 1.9 for *z* = 1, Xcorr ≥ 2.5 for *z* = 2, and Xcorr ≥ 3.5 for *z* = 3) and with mass accuracy better than 10 ppm. SRFs search result files were uploaded into the ProteoIQ software for parsing and spectral counting. Median relative intensity was compared between cases and controls using a non-parametric Wilcoxon rank sum test. Significance was set at the *p* value ≤ 0.05 level and no adjustments for multiple testing were performed.

### 3.6. Reverse-Transcription PCR

Total RNA was extracted following standard protocol of TRIzol^®^ RNA Reagents (Invitrogen). Genomic DNA contaminant was removed using DNA-free™ Kit (Ambion) containing RNase-free DNase I. Double-stranded cDNA was synthesized from 0.5 μg cleaned RNA with an olig-dT primer in a 20 μL Super Script III First-Stand Synthesis SuperMix reaction(Invitrogen) before further PCR or quantitative real-time PCR analyses. For the regular PCR, 3 μL supernatant was used as input DNA to process PCR reaction using GoTaq^®^ Hot Start Green Master Mix (Promega). The denatured primers were designed to amplify human RARRES1 and the sequences are: forward: 5-GAGCAGAG TTCAGTGTGCATG-3 and reverse: 5-CAACAAGAGGATTACCTGCTTTACAAG-3. Actin was used as an internal normalization reference and the sequences are, forward, 5-GAAGAGCTA TGAGCTGCCTGAC-3 and reverse, 5-AGGTCTTTACGGATGTCAACGT-3. 10 μL of PCR reaction were illuminated in 2% Ethidium bromide containing agarose gel.

Quantitative real time PCR 1 μL cDNA was used for real-time PCR in a 25 μL reaction. Monoplex real-time PCR was conducted in a 96-well spectrofluorometric thermal cycler (ABI PRISM 7900 Sequence detector system; Applied Biosystems). RARRES1 primers used for the real time PCR were chosen from PrimerBank as forward: 5-AGACAACAAGAGGATTACCTGCT-3 and reverse: 5-GCTGACTATTTCCAAGGGGTTT-3. Actin was used as the internal normalization control. Fluorescence was monitored during every PCR cycle at the annealing or extension step and during the post-PCR temperature ramp. Fold changes were then measured according to manufacturer instructions (Invitrogen).

### 3.7. Western Blot Analysis

RARRES1 expression was examined in both Schwann cell pellets and tissue specimens. After collecting the conditioned media for secretome analysis the remaining cells were washed three times with PBS and collected by gently scrapping into PBS media then pelleted by centrifugation at 300× g for 5 min. Total proteins were extracted from cell pellets in RIPA buffer composed of 25 mM Tris-HCl (pH 7.6), 150 mM NaCl, 1% NP-40, 1% sodium deoxycholate, 0.1% SDS (Thermo Scientific, Rockford, IL, USA). Equal amounts of total proteins (30 μg) from each sample were resuspended in equal volume of Laemmli buffer (Bio-Rad, Hercules, CA, USA). Proteins were separated by SDS-PAGE under reducing conditions and transferred to nitrocellulose membrane (GE Healthcare, Piscataway, NJ, USA) for Western blot analysis. Nonspecific binding sites were blocked with 5% milk (Bio-Rad) and membrane was incubated overnight with rabbit polyclonal antibody against human RARRES1 in 2.5% milk. We tested RARRES1 antibodies from two different companies: ab76530 from Abcam (Cambridge, MA, USA) and AF4255 from R&D Systems, Inc (Minneapolis, MN, USA). The next day, the membranes were rinsed with Tris buffer containing salt and Tween and incubated with goat anti-rabbit IgG (1:3K, Bio-Rad) conjugated with horseradish peroxidase. Immunoblots were visualized by chemiluminescence (GE Healthcare), and Ponceau red was used to control for protein loading.

### 3.8. Effect of All-Trans Retinoic Acid on the sNF96.2 Secreted RARRES1

Since the commercially available antibodies failed to detected and quantify secreted RARRES1, we synthesized stable isotope labeled RARRES1, to use as a standard, by culturing sNF96.2 cell line in DMEM culture media where ^12^C_6_-Arg and ^12^C_6_, ^14^N_2_-Lys were replaced by ^13^C_6_-Arg and ^13^C_6_, ^15^N_2_-Lys. After at least 7 cell doublings (roughly four passages), cellular proteins were uniformly labeled with these stable isotope-containing amino acids including secreted proteins. Cells were then washed 6 times with serum free media and incubated for another 24 hours in stable isotope labeled serum free media supplemented with ^13^C_6_-Arg and ^13^C_6_, ^15^N_2_-Lys. Conditioned media containing stable isotope labeled proteins was collected, centrifuged at 1000 × g and further filtered through a 0.2 μm Polyethersulfone syringe filter (Corning Inc.) then concentrated using 3 kDa MWCO centrifugal filtration device (Millipore Corporation, Billerica, MA, USA). The concentrated conditioned media was assayed for total protein content using Bio-Rad protein assay kit and stored at −20 °C until use. In parallel, the same cell line sNF96.2 was cultured in regular DMEM media supplemented with 10% FBS (ATCC), 100 U/mL of penicillin, 100 μg/mL of streptomycin (Invitrogen, Gibco, Carlsbad, CA, USA), and 1 mM of sodium pyruvate (Sigma Aldrich, St. Louis, MO, USA). When cells reached 80–90% confluence they were washed 6 times with serum free medium then incubated for another 24 hours in serum free medium supplemented with various doses of all-trans retinoic acid (0, 0.25, 0.5 and 1 μM). All-trans retinoic acid solution was freshly prepared in ethanol just before use. The untreated control cells were incubated with ethanol alone in serum free media for the same period of 24 hours. Conditioned media from untreated and treated cells were collected, spiked with equal amounts of condition media containing stable isotope labeled RARRES1 protein and processed as described above for SDS-PAGE and LC-MS/MS analysis. Bands corresponding to RARRES1 protein were sliced, in-gel digested by trypsin and the resulting peptides were analyzed by LC-MS/MS as described above. RARRES1 tryptic peptides were detected as pairs of stable isotope labeled and unlabeled peptides generated from the reference stable isotope labeled RARRES1 protein and the unlabeled RARRES1 protein secreted by the RA treated or untreated sNF96.2 cell cultures respectively. For quantification of RARRES1 protein in treated and untreated cells we used IP2 software, an integrated proteomic applications (http://integratedproteomics.com/) that uses the extracted ion chromatogram of labeled and unlabeled peptide pairs to determine the intensity ratios of light to heavy peptide and thus the relative quantities of secreted RARRES1 protein by RA treated and untreated sNF96.2 cells versus the spiked reference stable isotope labeled RARRES1 protein.

## 4. Conclusions

The secretome survey of cultured primary Schwann cells revealed differential secretion of several key proteins between NF1 derived Schwann cells and normal nerve derived Schwann cells. The majority of the differentially secreted proteins are involved in cell proliferation, adhesion and migration. Secretion of the tumor suppressor RARRES1 protein by NF1 is novel and merits further studies to characterize the structure and the function of this secreted RARRES1 protein.

Defining the role of these differentially secreted proteins in the context of Schwann cell biology might bring insight into Schwann cell transformation and neurofibroma pathogenesis. This will help define novel therapeutic strategies to slowdown or stop plexiform neurofibroma progression in patients diagnosed with NF1.

## Figures and Tables

**Table 1:** Collected neurofibroma specimens used to establish Schwann cell cultures.

Donor’s id	Age (years)	Gender	Tumor location	Diagnosis
NFSC 141	20	Female	Neck	Plexiform neurofibroma
NFSC 142	19	Male	Neck	Plexiform neurofibroma
NFSC 143R	17	Female	Right lumbar	Plexiform neurofibroma
NFSC 143L			Left lumbar	Plexiform neurofibroma

**Table 2:** List of proteins that were uniquely secreted by the plexiform neurofibroma Schwann cells or by the normal Schwann cells.

Accession *n*°	Protein name	NSC	NFSCs	Subcellular localization	Function
P49788	Retinoic acid receptor responder protein 1	0	45	Single-pass membrane	Tumor suppressor
P01024	Complement C3	0	43 *	secreted	complement pathway
P0C0L4	Complement C4-A	0	39	secreted	Complement pathway
P05090	Apolipoprotein D	0	15	secreted	Transporter
P22352	Glutathione peroxidase 3	0	10 *	secreted	Antioxidant
P36222	Chitinase-3-like protein 1	0	11 *	secreted	unknown
P55268	Laminin subunit beta-2	0	17	secreted	Cell attachment
P02649	Apolipoprotein E	0	7 *	secreted	Transporter
Q4LDE5	Sushi, von Willebrand factor type A	0	5	membrane/secreted	Cell attachment
Q92954	Proteoglycan 4	0	5	secreted	Adhesion inhibition
Q12805	EFEMP1	0	9 *	secreted	Cell adhesion/migration
P02771	Alpha-fetoprotein	0	6	secreted	Transporter
P55285	Cadherin-6	16	0	Membrane/cell surface	Cell adhesion
P24844	Myosin regulatory light polypeptide 9	12	0	cytoskeleton	Cytokenesis and cell locomotion
Q13308	Tyrosine-protein kinase-like 7	11	0	cell surface	Wnt signaling pathway
P09104	Gamma-enolase	9	0	cytosol/membrane	glycogenesis

NSC: normal human primary Schwann cells; NFSCs: plexiform neurofibroma derived Schwann cells. Numbers represent the average spectral count detected in either normal human Schwann cell group (*n* = 2) or plexiform neurofibroma derived Schwann cell group (*n* = 4).

* denote proteins that were secreted in 3 out of 4 NFSCs cultures;

EFEMP1: EGF-containing fibulin-like extracellular matrix protein 1.

**Table 3:** List of proteins that were differentially secreted between the plexiform neurofibroma and the normal Schwann cell cultures.

Accession *n*°	Protein name	NSC	NFSCs	Subcellular localization	Function
Q16363	Laminin subunit alpha-4	13	81	secreted	cell attachment/migration
Q14112	Nidogen-2	25	81	secreted	cell attachment/migration
P05155	Plasma protease C1 inhibitor	7	85	secreted	C1 complex inhibitor
P07585	Decorin	12	67	secreted	extracellular remodeling
Q7Z7G0	Target of Nesh-SH3	1	19	secreted	cell proliferation
P98160	Perlecan	5	46	secreted	extracellular matrix remodeling
P24592	Insulin-like growth factor-binding protein 6	4	16	secreted	regulates cell growth
P05121	Plasminogen activator inhibitor 1	727	284	secreted	inhibitor of fibrinolysis
P13611	Versican core protein	12	1	secreted	intercellular signaling and connecting cells with the extracellular matrix
Q6UVK1	Chondroitin sulfate proteoglycan 4	83	19	Cell surface	cell growth and migration
Q15262	Receptor-type tyrosine-protein phosphatase kappa	12	2	Cell surface	Negative regulator of EGFR signaling pathway
P05556	Integrin beta-1	13	1	Cell surface	Cell adhesion

NSC: normal human primary Schwann cells; NFSCs: plexiform neurofibroma derived Schwann cells. Numbers represent the average spectral count detected in either normal human Schwann cell group (*n* = 2) or plexiform neurofibroma derived Schwann cell group (*n* = 4).
